# Clinical validation of susceptibility-weighted cardiovascular magnetic resonance in comparison to T2 and T2* imaging for detection of intramyocardial hemorrhage following acute myocardial infarction

**DOI:** 10.1186/1532-429X-17-S1-P117

**Published:** 2015-02-03

**Authors:** Ananth Kidambi, John D Biglands, David M Higgins, David P Ripley, Arshad Zaman, David A Broadbent, Adam K McDiarmid, Peter P Swoboda, Tarique A Musa, Bara Erhayiem, John P Greenwood, Sven Plein

**Affiliations:** Multidisciplinary Cardiovascular Research Centre & Leeds Institute for Cardiovascular and Metabolic Medicine, University of Leeds, Leeds, UK; Philips Healthcare, Guildford, UK; Division of Medical Physics & Multidisciplinary Cardiovascular Research Centre, University of Leeds, Leeds, UK

## Background

Intramyocardial hemorrhage (IMH) identified by CMR is an established prognostic marker following acute myocardial infarction (AMI), but remains relatively underused in the clinical setting. Detection of IMH by T2-weighted or T2* CMR can be limited by long breath hold times and sensitivity to artefacts, especially at 3 Tesla. Alternative methods that can detect IMH with shorter breath hold times are therefore desirable. CMR is capable of detecting differences in the magnetic susceptibility of tissues. The paramagnetic properties of hemoglobin products within IMH cause local phase shifts relative to surrounding tissue. Phase data can be filtered and combined with magnitude data to generate susceptibility weighted MR images (SW MRI). SW imaging has been shown to be highly sensitive for the detection of cerebral hemorrhage, but typically uses long echo times, resulting in prolonged image acquisition. With the larger size of IMH, we hypothesized that SW MRI may be used with shorter echo times, leading to rapid imaging with breath hold times of approximately 4 seconds. We compared the image quality and diagnostic ability of this SW MRI pulse sequence with T2-weighted and T2* CMR to detect IMH at 3T.

## Methods

**Figure 1:**
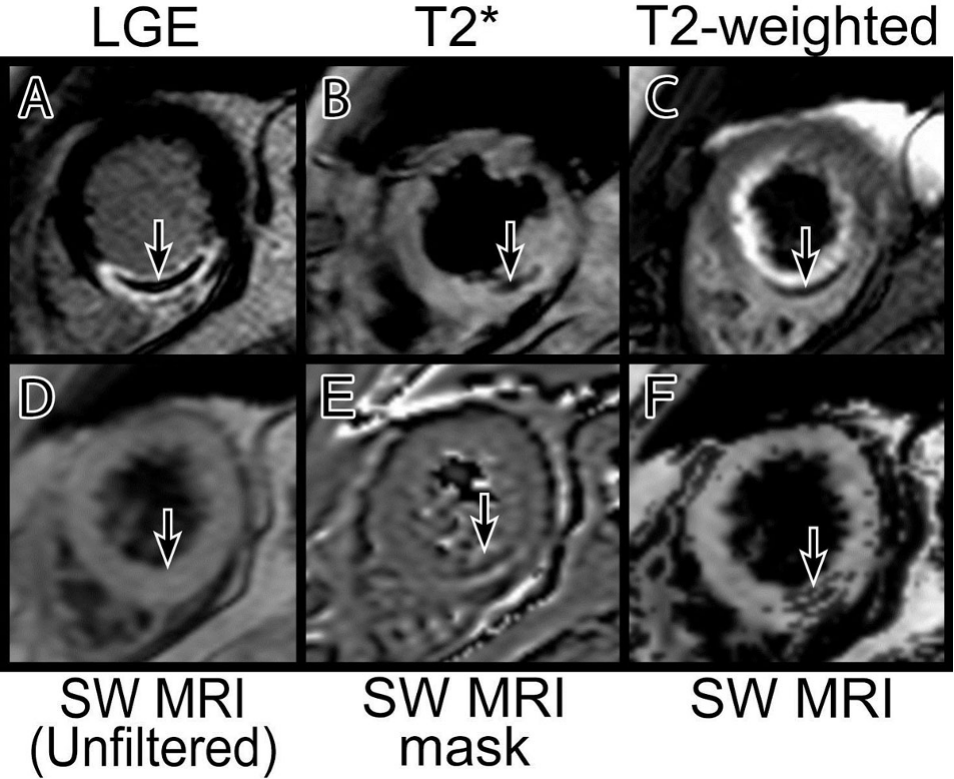
**Inferior AMI with IMH (arrowed).** Prior to phase enhancement, the SW MRI sequence has relatively low sensitivity for IMH (D). Application of the phase mask (E) results in clear visualization of IMH (F).

Forty-nine patients (42 males; mean age 58 years) underwent 3T CMR 2 days following reperfused AMI. T2-weighted, T2* and SW MRI images were obtained. Example images are shown in Figure 1. Signal and contrast measurements were compared between the three methods and the diagnostic accuracy of SW MRI was assessed against T2-weighted images by 2 independent, blinded observers. Image quality was rated on a 4-point scale from 1 (unusable) to 4 (excellent).

## Results

Of 49 patients, IMH was detected in 20 (41%) by SW MRI, 21 (43%) by T2-weighted and 17 (34%) by T2 star imaging (p=ns). Contrast to noise measurements were similar between the three sequences (β=-0.23, p=0.6 for SW MRI vs. T2-weighted and β=-0.69, p=0.8 for SW MRI vs. T2*). Compared to T2-weighted imaging, SW MRI had sensitivity of 93% and specificity of 86%. SW MRI had similar inter-observer reliability to T2-weighted imaging (Cohen's *κ* = 0.90 and *κ* = 0.88 respectively); both had higher reliability than T2* (*κ* = 0.53). Intra-observer reliability was *κ* = 0.79, *κ* = 0.79 and *κ* = 0.74 respectively for SW MRI, T2w and T2* imaging. Breath hold times were shorter for SW MRI (4 seconds vs. 16 seconds) with improved image quality rating (3.8±0.4, 3.3±1.0, 2.8±1.1 respectively; p<0.01).

## Conclusions

SW MRI at 3T can accurately and reproducibly identify areas of intramyocardial hemorrhage following acute myocardial infarction, with superior image quality to T2-weighted and T2* imaging and much shorter breath hold time.

## Funding

SP is funded by a British Heart Foundation fellowship (FS/1062/28409). DMH is an employee of Philips.

